# In vivo Drug Screening to Identify Anti-metastatic Drugs in *Twist1a-ER^T2^* Transgenic Zebrafish

**DOI:** 10.21769/BioProtoc.4673

**Published:** 2023-05-20

**Authors:** Joji Nakayama, Hideki Makinoshima, Zhiyuan Gong

**Affiliations:** 1 Tsuruoka Metabolomics Laboratory, National Cancer Center, Tsuruoka, Japan; 2 Shonai Regional Industry Promotion Center, Tsuruoka, Japan; 3 Department of Biological Science, National University of Singapore, Singapore; 4 Division of Translational Research, Exploratory Oncology Research, and Clinical Trial Center, National Cancer Center, Kashiwa, Japan

**Keywords:** In vivo drug screen, Metastasis, EMT, Twist1, Zebrafish

## Abstract

Here, we present an in vivo drug screening protocol using a zebrafish model of metastasis for the identification of anti-metastatic drugs. A tamoxifen-controllable *Twist1a-ER^T2^* transgenic zebrafish line was established to serve as a platform for the identification. By crossing *Twist1a-ER^T2^* with *xmrk* (a homolog of hyperactive form of the epidermal growth factor receptor) transgenic zebrafish, which develop hepatocellular carcinoma, approximately 80% of the double transgenic zebrafish show spontaneous cell dissemination of mCherry-labeled hepatocytes from the liver to the entire abdomen and tail regions in five days, through induction of epithelial to mesenchymal transition (EMT). This rapid and high-frequency induction of cell dissemination makes it possible to perform an in vivo drug screen for the identification of anti-metastatic drugs targeting metastatic dissemination of cancer cells. The protocol evaluates the suppressor effect of a test drug on metastasis in five days, by comparing the frequencies of the fish showing abdominal and distant dissemination patterns in the test drug–treated group with those in the vehicle-treated group. Our study previously identified that adrenosterone, an inhibitor for hydroxysteroid (11-beta) dehydrogenase 1 (HSD11β1), has a suppressor effect on cell dissemination in the model. Furthermore, we validated that a pharmacologic and genetic inhibition of HSD11β1 suppressed metastatic dissemination of highly metastatic human cell lines in a zebrafish xenotransplantation model. Taken together, this protocol opens new routes for the identification of anti-metastatic drugs.

Graphical overview

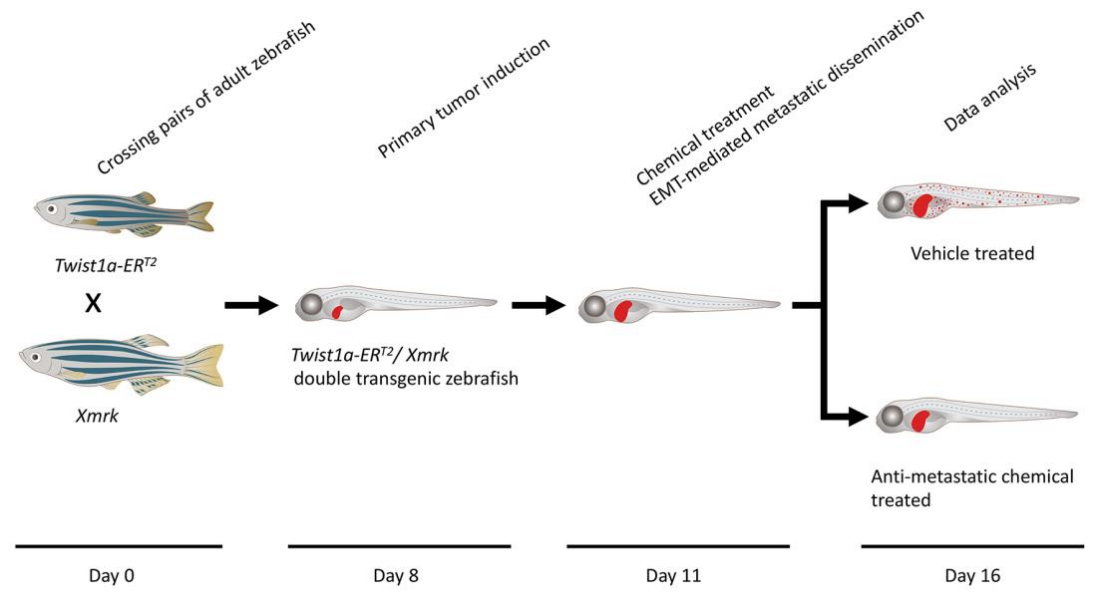

Timing

Day 0: Zebrafish spawning

Day 8: Primary tumor induction

Day 11: Chemical treatment

Day 11.5: Metastatic dissemination induction in the presence of a test chemical

Day 16: Data analysis

## Background

Metastasis is responsible for approximately 90% of cancer-associated mortality. It proceeds through multiple steps: invasion, intravasation, survival in the circulatory system, extravasation, colonization, and metastatic tumor formation in secondary organs with angiogenesis (Nguyen et al., 2009; Chaffer and Weinberg, 2011; Welch and Hurst, 2019). The dissemination of cancer cells is an initial step of metastasis, and its molecular mechanism involves a local breakdown of basement membrane, loss of cell polarity, and induction of epithelial to mesenchymal transition (EMT). EMT plays a central role in early embryonic morphogenesis; its process enables various types of epithelial cells to convert into mesenchymal cells, through a downregulation of epithelial markers such as E-cadherin and an upregulation of mesenchymal markers such as vimentin. Twist, a basic helix-loop-helix transcription factor, plays a critical role in inducing the EMT program (Tsai and Yang, 2013; Lu and Kang, 2019). Past studies showed that elevated expression of Twist is associated with poor survival rates in patients with cancer; also, ectopic expression of Twist confers metastatic properties on cancer cells through induction of EMT (Yang et al., 2004; Tsai et al., 2012).

**Figure 1.**
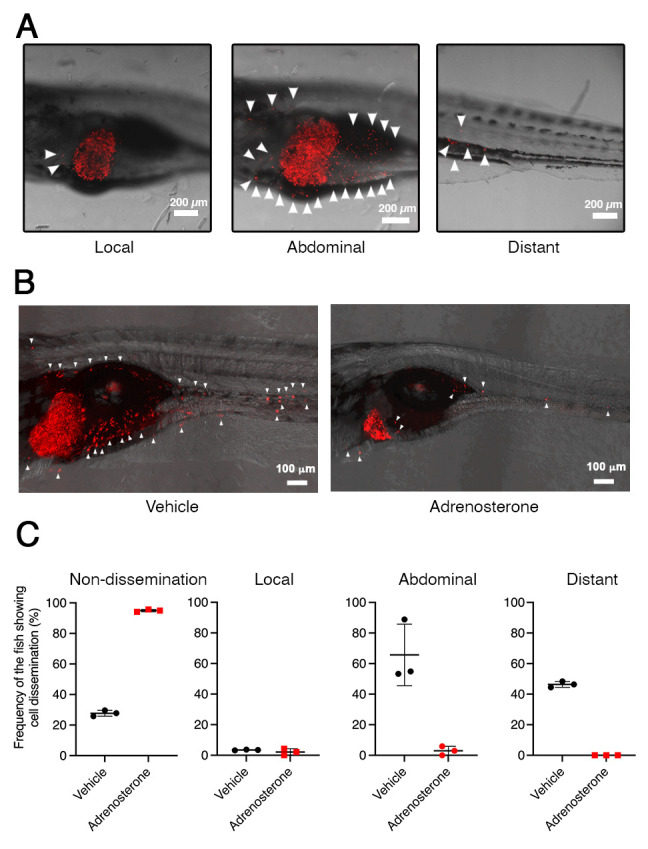
*Twist1a-ER^T2^/xmrk* double transgenic zebrafish offers an in vivo drug screening platform for the identification of anti-metastatic drugs. A. Representative images of the dissemination of mCherry-labeled hepatic cells from the liver in *Twist1a-ER^T2^/xmrk* double transgenic zebrafish at 16 days post-fertilization (dpf). The fish were treated with doxycycline and 4-hydroxytamoxifen (4-OHT). Some of the disseminated mCherry-positive cells are indicated by arrowheads. The images are shown as Z-stack images using 100× magnification. Scale bar, 200 μm. B. Representative images of the effect of adrenosterone on the dissemination of mCherry-positive cells in the fish at 16 dpf. Fish were treated with either vehicle (left) or adrenosterone (right). The images are shown as Z-stack images using 100× magnification. Scale bar, 100 μm. **C.** Mean frequencies of the fish showing the dissemination patterns of mCherry-positive cells in the vehicle- or adrenosterone-treated groups. Each value is presented as mean ± SEM from three independent experiments. Images are reprinted from Nakayama et al. (2020).

Cancer research using zebrafish as a model has attracted attention because this model offers many unique advantages that are not readily provided by other animal models (White et al., 2013; Osmani and Goetz, 2019). Furthermore, the zebrafish system has also been increasingly recognized as a chemical screening platform because it provides the advantage of high-throughput screening in an in vivo vertebrate setting with physiologic relevance to humans (Zon and Peterson, 2005; Letrado et al., 2018; Nakayama and Gong, 2020; Nakayama and Makinoshima, 2020; Nakayama et al., 2021b, 2022a and 2022b). Our study previously established a tamoxifen-controllable *Twist1a-ER^T2^* transgenic zebrafish line that serves as an in vivo drug screening platform for the identification of anti-metastasis drugs targeting metastatic dissemination of cancer cells. By crossing *Twist1a-ER^T2^* with *xmrk* (a homolog of hyperactive form of the epidermal growth factor receptor) transgenic zebrafish, which develop hepatocellular carcinoma, approximately 80% of the double transgenic zebrafish showed spontaneous cell dissemination of mCherry-labeled hepatocytes from the liver to the entire abdomen and tail regions in five days, through induction of an EMT (Nakayama et al., 2020; Lu et al., 2021). The dissemination patterns are generally divided into three categories: (i) local dissemination, in which disseminated mCherry-positive cells exist in close proximity to the liver; (ii) abdominal dissemination, in which the cells spread throughout the abdomen; and (iii) distant dissemination, in which the cells are observed over a broad region from the trunk to the tail (Figure 1A).

This rapid and high-frequency induction of cell dissemination makes it possible to perform an in vivo drug screen for the discovery of anti-metastasis drugs targeting metastatic dissemination of cancer cells. The protocol evaluates the suppressor effect of a test chemical through comparing the frequencies of the fish showing the abdominal and distant dissemination patterns in the test drug–treated group with those in the vehicle-treated group. Previous studies confirmed that ki16425 (a LPA1 inhibitor) or Y27632 (an inhibitor of Rho-associated coiled-coil-containing protein kinase), which have been reported to suppress metastasis in mice models of metastasis (Itoh et al., 1999; Boucharaba et al., 2006), could suppress cell dissemination in the fish model. In vivo drug screen using this model identified adrenosterone, an inhibitor for hydroxysteroid (11-beta) dehydrogenase 1 (HSD11β1), as having a potential to suppress metastatic dissemination of cancer cells (Figure 1B and 1C). Furthermore, pharmacologic and genetic inhibition of HSD11β1 were validated to suppress metastatic dissemination of highly metastatic human cell lines in a zebrafish xenotransplantation model (Nakayama et al., 2020 and 2021a). Taken together, our model offers an in vivo drug screening platform for the identification of anti-metastatic drugs.

## Materials and Reagents

1. 150 mm dish (Corning, catalog number: 430599)
2. 100 mm dish (Corning, catalog number: 430167)
3. 6-well flat-bottom plastic plates (Corning, catalog number: CLS3335)
4. Micron powder food (food for zebrafish larvae) (Sera)
5. 27 G needle tip (Terumo, catalog number: NN-2719S)
6. Plastic tea strainer (purchased from a local supermarket)
7. Transgenic zebrafish line Tg (*fabp10a:mCherry-T2A-Twist1a-ER^T2^*)
8. Transgenic zebrafish line Tg (*fabp10a:TA; TRE:xmrk; krt4:GFP*) known as *xmrk*
*Note: Tg (fabp10a:mCherry-T2A-Twist1a-ER^T2^) and Tg (fabp10a:TA; TRE:xmrk; krt4:GFP) are available upon request from Prof. Zhiyuan Gong, Department of Biological Sciences, National University of Singapore, Singapore.*
9. Doxycycline (Sigma-Aldrich, catalog number: D9891)
10. 4-hydroxytamoxifen (4-OHT) (Sigma-Aldrich, catalog number: H6278)
11. Methylcellulose (Sigma-Aldrich, catalog number: M7027)
12. Phenoxyethanol (Sigma-Aldrich, catalog number: 77699)
13. NaCl (Sigma-Aldrich, catalog number: S3014)
14. KCl (Sigma-Aldrich, catalog number: P9541)
15. MgSO_4_·7H_2_O (Sigma-Aldrich, catalog number: M2773)
16. CaCl_2_ (Sigma-Aldrich, catalog number: C4901)
17. Doxycycline stock solution (see Recipes)
18. 4-hydroxytamoxifen stock solution (see Recipes)
19. E3 medium (see Recipes)
20. 30% methylcellulose (w/v %) (see Recipes)
21. Phenoxyethanol (v/v %) (see Recipes)

## Equipment

1. Recirculating aquaculture system (Aquatic Habitat)
2. Fluorescence microscope (Olympus, catalog number: MVX10)
3. Leica TCS SP5X confocal microscope system (Leica)
4. Incubator for zebrafish embryos (AQUALYTIC, catalog number: 2418210)
5. External tank, a component of zebrafish breeding tank (Tecniplast, catalog number: ZB10BTE)
6. Perforated internal tank, a component of zebrafish breeding tank (Tecniplast, catalog number: ZB10BTI)
7. Polycarbonate divider, a component of zebrafish breeding tank (Tecniplast, catalog number: ZB10BTD)
8. Polycarbonate lid (Tecniplast, catalog number: ZB10BTL)

## Software

1. Image analysis software, Imaris 8 (Bitplane)

## Procedure

- **Zebrafish spawning (Day 0)**
  1. On the night before collecting the embryos, arrange pairs of male *xmrk* fish and female *Twist1a-ER^T2^* fish in a zebrafish breeding tank with a divider.
  2. Early in the morning, remove the divider to allow the fish to spawn.
  3. Scoop out the embryos with a plastic tea strainer.
  4. Transfer the embryos into a 150 mm plastic dish containing E3 medium.
  5. Remove dead embryos with a pipette.
  6. Maintain the embryos for six days in an incubator set at 27 °C.
  7. Change the E3 medium and remove dead embryos with a pipette daily.
*Notes:*
  - *Adult Twist1a-ER^T2^ and xmrk transgenic zebrafish lines were maintained in a recirculating aquaculture system with an ambient water temperature of approximately 28 °C in Singapore. 6–8 pairs of female heterozygous* Twist1a-ER^T2^ *transgenic fish and male heterozygous* xmrk *transgenic fish were crossed in the breeding tank (one pair per tank). Young adult zebrafish (3–9 months of age) were preferentially used for the crossing. Electric lighting in the facility was switched on from 07:00 to 19:00. Brine shrimps were fed to both zebrafish lines five times a day, at 08:30, 10:30, 12:30, 14:30, and 16:30.*
  - *The Fish facility at the National University of Singapore connects to the outdoors. Therefore, the facility does not have air conditioning and the room temperature in the facility fluctuates between 25 °C and 30 °C. Embryos and larvae are maintained in an incubator set at 27 °C. Therefore, all larvae zebrafish were maintained at 27 °C to ensure consistency of the zebrafish development.*
  - *If a specific developmental stage is required, the embryos should be grown at the optimal condition of 28.5 °C, in order to allow a standard development of the embryos and larvae (Westerfield, 2007).*
  - *Feeding of the zebrafish larvae began at 5 dpf. A pinch of micron powder was diluted in 1 mL of E3 medium and added to the zebrafish larvae every morning. The zebrafish larvae were transferred into each well of a 6-well plate at a time point between 8 and 11 dpf, and a few drops of the E3 medium were added to each well every morning.*
- **Collect *Twist1a-ER^T2^/xmrk* double transgenic zebrafish (Day 6)**
  1. Transfer the zebrafish larvae into a 50 mL tube.
  2. To anesthetize the fish, add 2% (v/v) phenoxyethanol to the E3 medium with the micron powder that contains the fish. Thus, the final concentration of phenoxyethanol is 0.02%.
  3. Array the fish on a lid of a 150 mm plastic dish.
  4. Visualize under a fluorescence microscope and use a pipette to collect the fish expressing green fluorescent protein (GFP) in the skin (Figure 2).
    **Figure 2.**
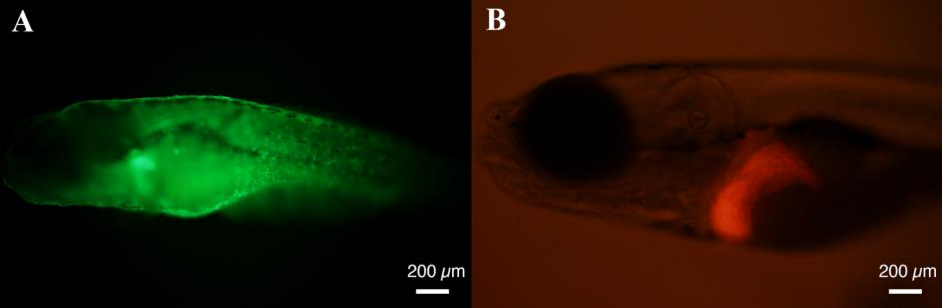
A *Twist1a-ER^T2^/xmrk* double transgenic zebrafish expresses green fluorescent protein (GFP) in a skin-specific manner (A) and mCherry in a liver-specific manner (B). Representative images of GFP and mCherry signals in *Twist1a-ER^T2^/xmrk* double transgenic zebrafish at 6 days post-fertilization (dpf). Scale bar, 200 μm.
  5. Among the GFP-positive fish, visualize under a fluorescence microscope and use a pipette to collect those expressing mCherry in the liver (Figure 2).
  6. Transfer the collected fish into a 150 mm plastic dish containing E3 medium.
  7. Maintain the fish for two days in an incubator set at 27 °C.
*Notes:*
  - *At 6 dpf, Tg (*fabp10a:mCherry-T2A-Twist1a-ER^T2^*) begins to express the gene coding mCherry-T2A-Twist1a-ER^T2^ in a liver-specific manner. Tg (*fabp10a:TA; TRE:xmrk; krt4:GFP*) expresses GFP in a skin-specific manner. Therefore*, Twist1a-ER^T2^/xmrk *double transgenic zebrafish is indicated as mCherry- and GFP-positive in the liver and skin, respectively.*
  - *The screening process is divided into two steps. Firstly, zebrafish possessing the* xmrk *transgene are screened at 3–5 dpf and then the* xmrk *transgenic zebrafish possessing the* Twist1a-ER^T2^ *transgene are screened at 6 dpf.*
  - *The zebrafish larvae should not be overcrowded in the 150 mm plate, since overcrowding negatively affects their viability. By 6 dpf, 100–300 zebrafish larvae can be maintained in the 150 mm plate. After 6 dpf, a maximum of 100 zebrafish larvae should be maintained in the 150 mm plate.*
- **Primary tumor induction (Day 8)**
  1. Aliquot a maximum of 100 zebrafish larvae into a 150 mm plate along with 100 mL of E3 medium.
  2. At 8 dpf, treat the fish with 30 μg/mL of doxycycline in E3 medium for three days.
  3. Change the E3 medium containing doxycycline every two days.
*Note: 100 μL of doxycycline stock solution (30 mg/mL) is added to 100 mL of E3 medium.*
- **Chemical treatment (Day 11)**
  1. Aliquot approximately 20 zebrafish larvae into each well of a 6-well plate with 8 mL of E3 medium containing doxycycline (30 μg/mL) at a time point between 8 and 11 dpf.
  2. Add each test chemical to each well of the plate at a final concentration of 5 μmol/L.
  *Notes*:
  - *All test chemicals are dissolved in DMSO at a concentration of 10 mmol/L (stock solution).*
  - *4 μL of each stock solution (10 mmol/L) is diluted with 100 μL of E3 medium and added into each well of the plate containing 8 mL of E3 medium.*
- **Metastatic dissemination induction in the presence of a chemical (Day 11.5)**
  1. Beginning 12 h after the addition of the test chemical, treat the fish with 0.1 μmol/L of 4-OHT in E3 medium for five days.
  2. Change the E3 medium containing doxycycline, 4-OHT, and the test chemical every two days.
*Notes:*
  - *The quality of the E3 medium is critical for the survival of the zebrafish larvae. The fish excrete waste materials including urine. Waste materials and leftover foods are harmful for the viability of the fish. Thus, the E3 medium should be changed before a high concentration of waste materials is reached.*
  - *We recommend changing the E3 medium every two days after 8 dpf.*

## Data analysis

Timing: Day 16

1. At five days after the first 4-OHT addition, transfer the zebrafish larvae into a 50 mL tube.
2. To anesthetize the fish, add 160 μL of 2% phenoxyethanol (v/v) into E3 medium containing the fish. The final concentration of phenoxyethanol is 0.02% (v/v) in a total volume of 16 mL.
3. Array the fish on the lid of a 100 mm plastic dish.
4. Embed the fish with a drop of 30% methylcellulose.
5. Manually orient the fish into a lateral view using a 27 G needle tip.
6. Determine the pattern of cell dissemination in the fish under a fluorescence microscope.
7. Count the number of fish showing each dissemination pattern of mCherry-labeled cells from the liver.
8. For taking images, capture serial sections of the fish in 8 μm Z-step intervals using a Leica TCS SP5X confocal microscope system. Process Z-stack images using the image analysis software Imaris (Bitplane).
*Notes:*

- *The dissemination patterns are generally divided into three categories: (i) local dissemination, in which disseminated mCherry-positive cells exist in close proximity to the liver; (ii) abdominal dissemination, in which the cells spread throughout the abdomen; and (iii) distant dissemination, in which the cells are observed over a broad region from the trunk to the tail (Figure 1).*
- *The suppressor effect of a test drug is evaluated by comparing the frequencies of the fish showing the abdominal and distant dissemination patterns in the test drug–treated group with those in the vehicle-treated group.*
- *The dissemination patterns are classified by viewing the fish under a fluorescence microscope. To avoid bias, the experimenter should be blinded to the treatments.*

**Limitations**

There is a limitation on the number of *Twist1a-ER^T2^/xmrk* double transgenic zebrafish that can be prepared (a few hundred). This limitation determines how many chemicals can be tested in one screening session. To test one chemical, approximately 20 double transgenic fish are needed. Following Mendel's laws, the rate of the double transgenic fish production is 25% when a heterozygous *Twist1a-ER^T2^* transgenic zebrafish is crossed with a heterozygous *xmrk* transgenic zebrafish. If 20 chemicals are to be tested, at least 400 double transgenic fish would be required. To prepare 400 double transgenic fish, pairs of *Twist1a-ER^T2^* and *xmrk* fish would need to generate approximately 2,000 embryos. Thus, when following the protocol described above, a maximum of 20 test chemicals can be evaluated.

## Recipes

1. **Doxycycline stock solution**
  **Table:** 

Reagent	Final concentration	Amount
Doxycycline	50 mg/mL	500 mg
ddH_2_O	n/a	10 mL
Total	n/a	10 mL
2. **4-hydroxytamoxifen stock solution**
  **Table:** 

Reagent	Final concentration	Amount
4-OHT	100 mM	387 mg
Ethanol	n/a	1,000 mL
Total	n/a	1,000 mL
3. **E3 medium**
  **Table:** 

Reagent	Final concentration	Amount
NaCl	5.0 mM	0.292 g
KCl	0.17 mM	0.013 g
MgSO_4_·7H_2_O CaCl_2_ ddH_2_O Total	0.33 mM 0.33 mM n/a	0.081 g 0.048 g 1,000 mL 1,000 mL
4. **30% methylcellulose (w/v %)**
  **Table:** 

Reagent	Final concentration	Amount
Methylcellulose	30%	30 g
ddH_2_O	n/a	100 mL
Total	n/a	100 mL
5. **Phenoxyethanol (v/v %)**
  **Table:** 

Reagent	Final concentration	Amount
Phenoxyethanol	0.02%	20 μL
ddH_2_O	n/a	100 mL
Total	n/a	100 mL
